# Supplementary material for: Mutations in PCYT2 disrupt etherlipid biosynthesis and cause a complex hereditary spastic paraplegia
Source: Brain. 2019 Oct 22;142(11):3382–97. doi: 10.1093/brain/awz291 (PMC6821184; doi:10.1093/brain/awz291)
Supplement: awz291_Supplementary_Data [file awz291_supplementary_data.zip › awz291-Suppl_data/Supplementary_Data4.zip!/Supplementary information 3 - fibroblast lipidomics.zip!/Fibroblast lipidomics/Controle vs Patient1/Statistical plots and data/Cer[d] species/Volcano plot_alternative.pdf]

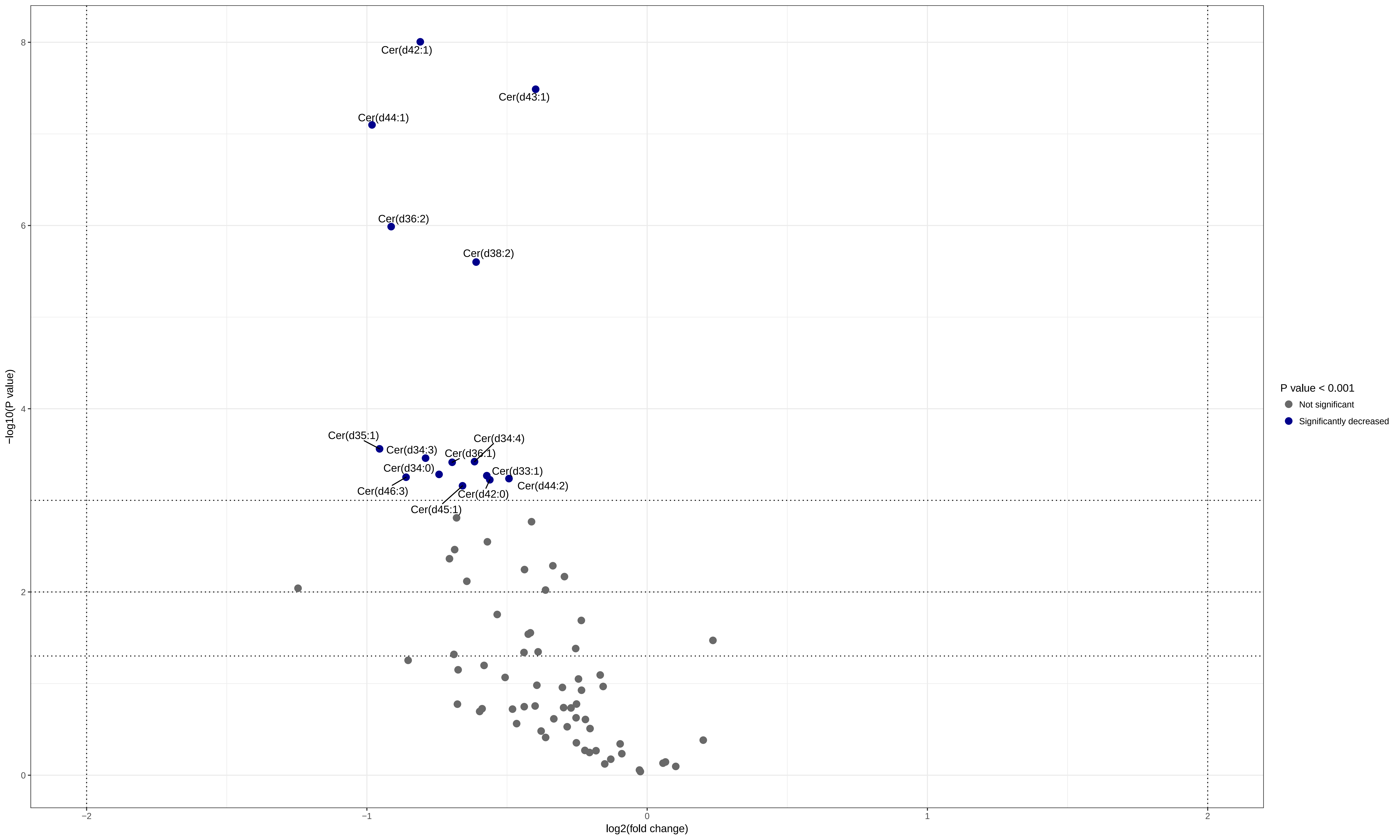

Cer(d42:1)
Cer(d43:1)
Cer(d44:1)
Cer(d36:2)
Cer(d38:2)
Cer(d35:1)
Cer(d34:3)
Cer(d34:4)
Cer(d36:1)
Cer(d34:0)
Cer(d33:1)
Cer(d44:2)
Cer(d46:3)
Cer(d42:0)
Cer(d45:1)
8
6
4
2
0
−2
−1
0
1
2
−log10(P value)
log2(fold change)
P value < 0.001
Not significant
Significantly decreased
